# Neonatal hypothermia and associated factors within six hours of delivery in eastern part of Ethiopia: a cross-sectional study

**DOI:** 10.1186/s12887-019-1632-2

**Published:** 2019-07-24

**Authors:** Wubet Alebachew Bayih, Nega Assefa, Merga Dheresa, Biniam Minuye, Solomon Demis

**Affiliations:** 1 Department of Nursing, College of Health Sciences, Debre Tabor University, P.O.BOX 272 Debre Tabor, Ethiopia; 2 College of Health and Medical Sciences, Haramaya University, P.O.BOX 235 Harar, Ethiopia

**Keywords:** Hypothermia, Thermo-regulation, Thermal care, Haramaya University

## Abstract

**Background:**

Neonatal hypothermia plays a significant role in increasing neonatal death by 80% for every 1 degree Celsius decrease of body temperature, especially in sub Saharan countries. A global burden of neonatal hypothermia indicated that 53% of Ethiopian newborns developed hypothermia due to different socio-demographic, behavioral, physiological and birth context related factors. However, the significance of these factors along the spectrum of public health institutions in the study area hasn’t been yet studied.

**Objective:**

To assess the prevalence and associated factors of neonatal hypothermia within six hours of delivery at public health institutions of Harar city, Eastern Ethiopia, 2018.

**Methods:**

An institution based cross sectional study was conducted at Harar city after stratified followed by random selection of 3 public health institutions. Every other eligible newborn was included by systematic sampling to yield a sample of 403 newborns and their axillary temperature was measured by a calibrated digital thermometer within six hours of delivery from January 25 to February 19, 2018. A pre-tested anonymous questionnaire and checklist were used. The collected data were cleaned, coded and entered into Epi -data version 4.2 and exported to STATA version 12. Binary logistic regression model was considered and those variables with *P* < 0.25 in the bivariable analysis were included in to final model after which statistical significance was declared at *P* < 0.05. The goodness of fit was tested by Hosmer-Lemeshow statistic and Omnibus tests. Multi co-linearity was diagnosed using standard error and correlation matrix.

**Results:**

The prevalence of neonatal hypothermia in the study area was 66.3% (95% CI: 61.1, 70.5%). No skin to skin contact (AOR = 2.87, 95% CI:1.48, 5.57), no wearing cap (AOR = 2.10, 95% CI:1.17, 3.76), no warm intra-facility transportation (AOR = 3.18, 95% CI: 1.84, 5.48), born to mothers having obstetric complication (AOR = 2.42, 95% CI:1.28, 4.57), prematurity (AOR = 3.37, 95% CI:1.53, 7.44) and neonatal health problem (AOR = 4.24, 95% CI:1.92, 9.34) were significantly associated with hypothermia.

**Conclusion:**

The prevalence of neonatal hypothermia was relatively high. Therefore, adherence should be made to the thermal care mainly the cost effective ones like wearing cap, skin to skin contact and warm transportation.

## Background

According to World Health Organization, neonatal hypothermia is defined as an abnormal thermal state in which the newborn’s body temperature is below 36.5 °C [1]. It is classified into three different categories based on core temperature of a new-born below 36.5 °C measured as skin temperature in the axilla: mild hypothermia (36.0 °C–36.4 °C), moderate hypothermia (32.0 °C–35.9 °C) and severe hypothermia (< 32.0 °C) [1–3]. For this study, hypothermia was defined as the newborn’s body temperature < 36.5 °C.

Immediately after birth, neonates can lose heat through conduction, convection, evaporation and radiation that can cause a reduction of up to 2 °C of their body temperature within the first 10–20 min of birth [1]. Evaporative loss forms the major route of heat loss in the immediate newborns [1–4]. Therefore, if no action is taken immediately after delivery, the core and skin temperatures of a term neonate can decrease at a rate of approximately 0.1°c and 0.3°c per minute respectively due to their risky characteristics of large surface area per unit body weight [5, 6], decreased subcutaneous fat, greater body water content, immature skin and poorly developed thermoregulatory mechanism [1, 7, 8]. Morbidity and mortality of newborns is uniformly higher among premature and low birth weight newborns as compared to their counter parts [9].

It is a common cause of neonatal morbidity and mortality, even in warmer tropical countries [4] and hence increasingly recognized as a risk factor for new-born survival [5]. Neonatal hypothermia is a worldwide problem with higher prevalence rate among low resource settings. In sub-Saharan countries, it increases neonatal death by 80% for every 1 degree Celsius decrease of body temperature because it affects all neonatal body systems [3]. It is a major co-morbid of prematurity, severe infections and asphyxia contributing much for the smallest decline in neonatal mortality rate of the Eastern Mediterranean and the African regions [12]. A global burden of neonatal hypothermia indicated that 53% of newborns in Ethiopia developed hypothermia due to different socio-demographic, physiologic, contextual and behavioral factors [5, 6, 13–28]. However, the significance of these factors within six hours of delivery along the spectrum of public health institutions in the study area hasn’t been yet studied.

## Methods

### Study area and period

This study was held in the capital city of Harari regional state, Harar, Eastern Ethiopia which is 525 km far from Addis Ababa and 48 km away from Dire Dawa city. The institutional delivery coverage of the region was 79%.There were 6 public health institutions that provide delivery service to the community [29]. The public health institutions in the city were first stratified into strata of specialized Hospital, General Hospital and Health center after which one specialized Hospital namely Hiwot Fana Specialized University Hospital, one General Hospital namely Jugal General Hospital and one Health Center namely Jenniela Health Center were selected randomly from each stratum respectively. The study was conducted from January 25/2018 until February 19/2018.

### Study design and participants characteristics

A descriptive institutional based quantitative cross-sectional study design was conducted. All newborns that were within six hours of their delivery at public health institutions of Harar city together with their mothers were included in the study. However, abandoned newborns were excluded of the study because without maternal involvement it wasn’t possible to address most of the variables in the study.

### Sample size determination and sampling procedure

The desired sample was determined by using double population proportion formula with the assumptions of 95% CI, 5% margin of error, 80% power, exposed to unexposed ratio 1:1, 10% non-response rate and an important variable of the study (skin to skin contact(P1): 75% and Didn’t have skin to skin contact(P2): 87%) [22]. The final sample size used for the study was 403. By using systematic random sampling, every other eligible live newborn was included into the study.

### Measurement and data collection procedure

Data collection was done by five diploma midwives after they were given one day training of data collection procedure. Data were collected carefully using a pretested and structured interviewer administered questionnaire that contained maternal socio-demographic characteristics and behavioral factors like skin to skin contact, breast feeding, immediate drying, newborn wrapping, cap wearing and warm intra-facility newborn transportation. Moreover, a checklist was used to obtain factors including maternal obstetric complication, neonatal age, birth weight, gestational age at birth, mode of delivery, time of delivery, APGAR score, resuscitation history and neonatal health problem. Axillary temperature of newborns was measured within six hours of delivery for 3 min of duration by using digital thermometer at either delivery or maternity ward.

### Data quality control

The data collection tool was adopted and modified from studies conducted in Ethiopia, Nigeria, Tanzania and Uganda which passed through peer review for its validity and published [16–18, 22, 24]. Moreover, a calibrated thermometer (model of MT-101 MT-111) that had measurement accuracy of ±0.1 °C for the temperature range of (35.5–42.9 °C) and ± 0.2 °C for the temperature range of (32.0–35.5 °C or above 42 °C) was used [24]. The data collection tool was pretested just two weeks prior to the actual data collection using 20 newborns at the study area based on which some modifications were made to the originally prepared tool. One day training and clear orientation was provided for data collectors and supervisors on the process of data collection. During data collection, data collectors were closely monitored and guided by two BSC nurse supervisors for complete and appropriate collection of the data and reporting to the principal investigator was done on a daily basis. **The collected** data were double entered into Epidata version 4.2 by two data clerks for validation purpose. The entered data were multivariate analyzed for statistical adjustment of possible confounders.

### Operational definition

#### Hypothermia

A newborn was considered to be hypothermic when its axillary temperature was less than 36.50C [1, 28].

#### Immediate drying

A newborn was considered to be immediately dried if and only if its skin was dried on maternal abdomen as soon as delivery prior to cord cutting using dry and pre-warmed towel [1].

#### Neonatal health problem

Refers to presentation of the neonate with any problem that can trouble its health (congenital malformation, asphyxia, jaundice, respiratory distress, bleeding disorder, meconium aspiration syndrome, etc.) [1, 28].

#### Proper wrapping

A newborn was considered as properly wrapped if and only if itswhole body including the head and the limbs were wrapped with the use of a pre-warmedand dry towel [1].

#### Warm intra-facility transportation

A newborn was considered as warmly transported if and only if it was taken from delivery unit to postnatal or neonatal intensive care unit while being in direct contact with its mother’s skin [1].

### Data processing and analysis

The collected data were cleaned manually, coded and entered into Epi data version 4.2 and exported to STATA Version 12 statistical software for data transformation and further analysis. Descriptive statistics like frequencies, proportion, and summary statistics (mean and standard deviation) were used to describe the study population in relation to relevant variables and presented in tables and graphs. Multi-collinearity between the study variables was diagnosed using standard error and correlation matrix. The assumptions for binary logistic regression model were first checked and then bivariate analysis was carried out to identify candidate variables (*p* < 0.25) for multivariate analysis. Using these candidate variables, multivariate analysis was performed to investigate statistically significant independent predictors of hypothermia by adjusting for possible confounders. Finally, variables whose p- value less than 0.05 (*p* < 0.05) from multivariate analysis were declared as statistically significant. Adjusted odds ratio with 95% CI was considered to identify the strength of association between neonatal hypothermia and its predictors.

### Ethical consideration

Ethical approval was obtained from the Institutional Health Research Ethics Review Committee of College of Health and Medical Sciences of Haramaya University. Official letter was obtained from Haramaya University and submitted to each Hospital and health center to get permission from the respective directors of the institutions. Before conducting the interview, an informed and voluntarily signed written consent (thumb print for those unable to write) was obtained from all the eligible mothers of the newborns. The thermometer was disinfected by 70% ethyl alcohol disinfectant with a damp cloth after every measure of axillary temperature of the newborn to prevent infection transmission. Hypothermic premature newborns and those with associated problems were helped for referral to neonatal intensive care unit for better management whereas those mildly and moderately hypothermic ones without medical problem were helped by rehabilitative thermal care measures like proper wrapping, frequent breast feeding, skin to skin contact, closing doors and windows etc. Moreover, mothers were advised of thermal care measures on the way to their home and while at their home.

## Results

### Socio-demographic factors

A total of 403 mothers with their neonates were included in the study making 100% response rate. The mean maternal age was 32 years (SD = ±5.5) and more than half of the mothers (51.9%) were in the age group between 20 and 34 years of age. Two hundred twenty nine (56.8%) were rural residents. One hundred thirty two respondents (32.8%) were unable to read and write and 119(29.5%) of the respondents were farmers. Furthermore, 258 respondents (64%) were multiparous (Table 1).

**Table 1:** Socio-demographic characteristics of mothers who gave alive birth at public health institutions of Harar city, Eastern Ethiopia, 2018

Variables (***n*** = 403)	Category	Frequency	Percentage (%)
**Residence**	Urban	174	43.2
	Rural	229	56.8
**Ethnicity**	Oroomo	155	38.5
	Amhara	108	26.8
	Harari	58	14.4
	Guraghe	48	11.9
	Tigray	28	6.9
	Other	6	1.5
**Religion**	Muslim	178	44.2
	Orthodox	134	33.3
	Protestant	84	20.8
	Other	7	1.7
**Age**	< 20	12	3
	20–34	208	51.6
	35–49	183	45.4
**Educational status**	Unable to read and write	132	32.8
	Primary education	131	32.5
	Secondary education	37	9.2
	Diploma and above	103	25.6
**Occupational status**	Housewife	54	13.4
	Government employee	104	25.8
	Private employee	116	28.8
	Student	10	2.5
	Farmer	119	29.5
**Parity**	Primiparous	145	36
	Multiparous	258	64

### Behavioral factors

Three hundred four (75.4%) of the newborns were born to mothers who had antenatal care. Almost all of the newborns, 390(96.8%), were immediately dried. More than half of the newborns, 207 (51.4%), weren’t wrapped properly. Nearly three-fourth of the newborns, 301 (74.7%) weren’t made in contact with their mother’s skin. One hundred eighty seven newborns (46.4%) didn’t wear cap. Breastfeeding was initiated within one hour of birth for 287 newborns (71.2%) and 251(62.3%) newborns weren’t warmly transported from delivery ward to post natal or NICU (Table 2).

**Table 2:** Behavioral factors of hypothermia among newborns that were born at public health institutions of Harar cty, Eastern Ethiopia, 2018

Variable (n = 403)	Category	Frequency	Percentage (%)
Had antenatal care	Yes	304	75.4
	No	99	24.6
Immediate drying	Yes	390	96.8
	No	13	3.2
Skin to skin contact	Yes	102	25.3
	No	301	74.7
Proper wrapping	Yes	196	48.6
	No	207	51.4
Wearing cap	Yes	216	53.6
	No	187	46.4
Breastfeeding within an hour of birth	Yes	287	71.2
	No	116	28.8
Warm intra-facility newborn transportation	Yes	152	37.7
	No	251	62.3

### Birth context related factors

It was during the day time that more than half of the newborns, 213(52.9%), were delivered. Furthermore, 257(63.8%) of the newborns were delivered through spontaneous vaginal delivery. Most of the newborns were delivered at the specialized University Hospital (Table 3).

**Table 3:** Birth context related factors of hypothermia among newborns that were born at public health institutions of Harar city, Eastern Ethiopia, 2018

Variable (n = 403)	Category	Frequency	Percentage (%)
Delivery time	Day time	213	52.9
	Night time	190	47.1
Delivery mode	SVD	257	63.8
	Instrumental	34	8.4
	C/S	112	27.8
Place of delivery	Health Center	48	11.9%
	General Hospital	105	26.1%
	Specialized Hospital	250	62.0%

### Newborn physiology related factors

The average neonatal age was 3.37 h (SD = ±1.72) while the average birth weight was 2.94Kg (SD = ±0.63). Moreover, the average gestational age at birth was almost 38 weeks (SD = ±2.52) with an average fifth minute APGAR score of 7 (SD = ±1.60). Male newborns, 202 (50.1%), were almost equal to females, 201(49.9%). Majority of the newborns, 347(86.1%), were born single. One hundred four (25.8%) of the total newborns were born with health problems. From the overall newborns, one hundred nineteen (29.5%) were born to mothers who had obstetric complication during pregnancy or labor and/delivery. From these complications, preeclampsia/eclampsia disorders comprises the highest percentage, 63(52.9%), followed by PROM/Sepsis 23(19.3%), APH, 19(16%), Obstructed/prolonged labor, 12(10.1%) and PPH, 2(1.7%) respectively. Forty (9.9%) of the newborns were given Cardio-Pulmonary Resuscitation after their birth (Table 4).

**Table 4:** Physiological factors of hypothermia among newborns that were born at public health institutions of Harar city, Eastern Ethiopia, 2018

Variable (n = 403)	Category	Frequency	Percentage (%)
Sex	Male	202	50.1
	Female	201	49.9
Age (hours)	(0–2]	142	35.2
	(2–4]	119	29.5
	(4–6]	142	35.2
Number of newborns	Single	347	86.1
	Twin	56	13.9
Birth weight (Kg)	< 2.5 (low birth weight)	81	20.1
	≥2.5	322	79.9
Gestational age at birth (weeks)	< 37 weeks (preterm)	99	24.6
	≥37 weeks	304	75.4
APGAR score	Low	25	6.2
	Moderate	99	24.6
	Normal	279	69.2
Neonatal medical problem	Yes	104	25.8
	No	299	74.2
Maternal obstetric complication	Yes	119	29.5
	No	284	70.5
Cardio-pulmonary resuscitation given	Yes	40	9.9
	No	363	90.1

### Prevalence of neonatal hypothermia

**Fig. 1:**
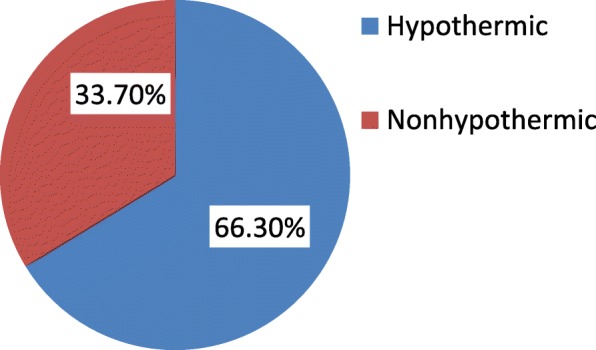
Prevalence of hypothermia among newborns that were within six hours of delivery at public health institutions of Harar city, 2018

**Fig. 2:**
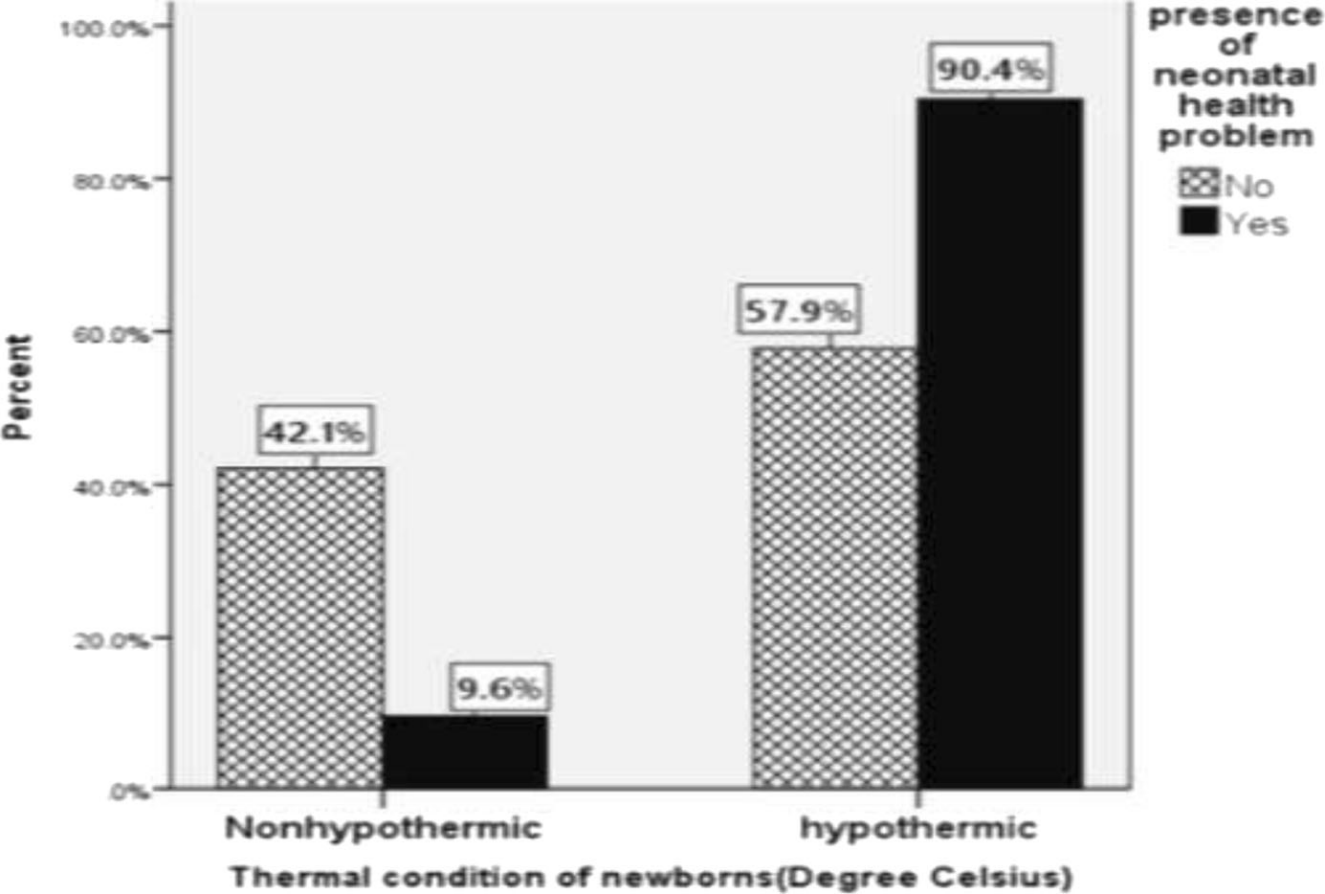
Comparison of hypothermia with respect to the presence of neonatal health problem among newborns who were delivered at public health institutions of Harar city, Eastern Ethiopia, 2018

**Fig. 3:**
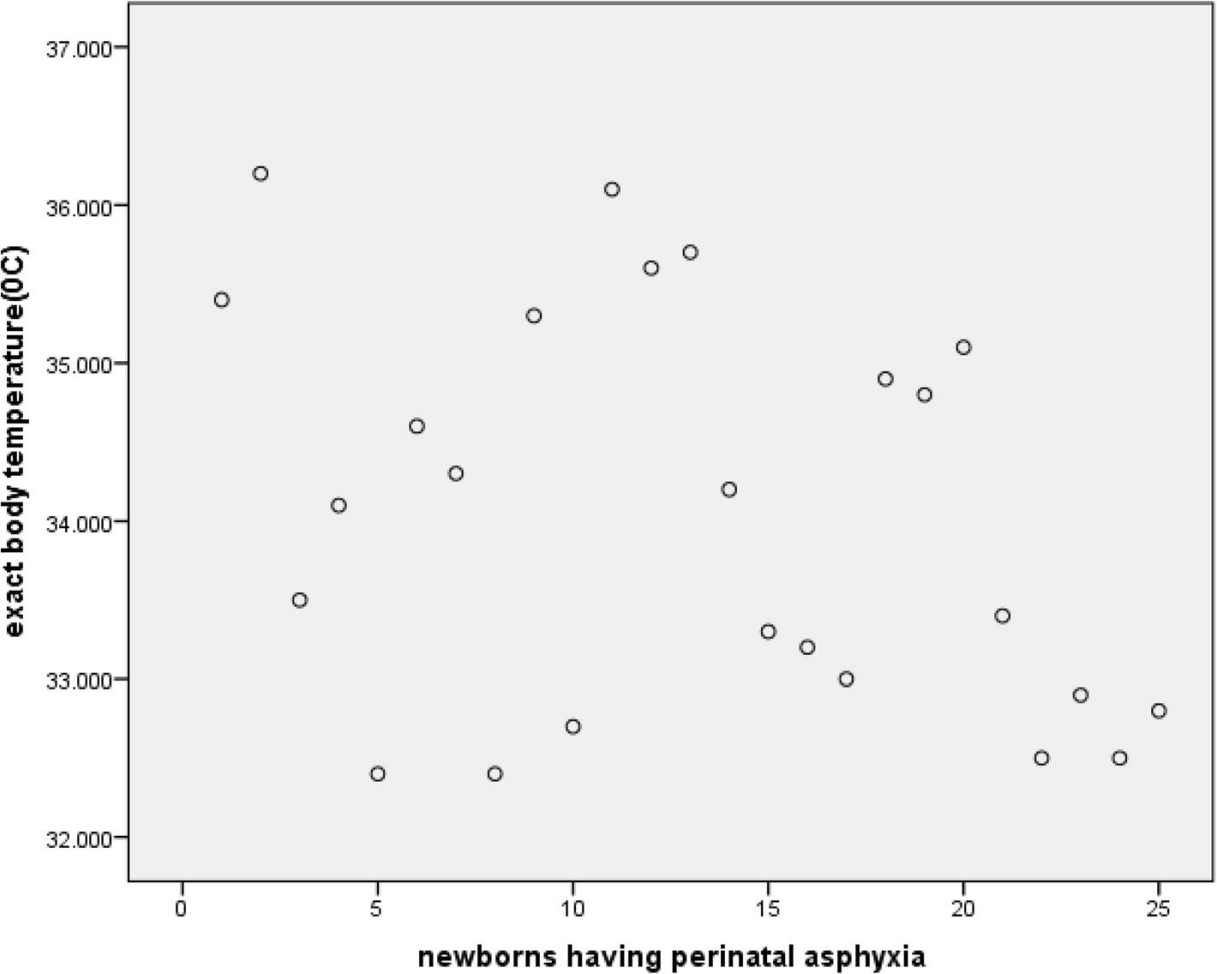
Scatter plot of the exact body temperature of perinatally asphyxiated newborns who were delivered at public health institutions of Harar city, Eastern Ethiopia, 2018

The prevalence of neonatal hypothermia among newborns who were delivered at public health institutions of Harar city was 267 (66.3%) [95% CI: 61.1, 70.5%)] with mean axillary temperature of 36.24 degree Celsius (SD = ±0.69) (Fig. 1). Majority of the hypothermic newborns, 150(56.2%), were mildly hypothermic whereas the rest 117(43.8%) were moderately hypothermic. The prevalence of hypothermia was highest, 94(90.4%), among newborns that had health problem(s) (Fig. 2). These problems were respiratory distress syndrome 39(37.5%), hypoglycemia 33(31.7%), perinatal asphyxia 25(24.0%), meconium aspiration syndrome 16(15.4%), congenital malformation 14(13.5%), early onset neonatal sepsis 12(11.5%) and jaundice 8(7.7%). Moreover, hypothermia was found to be more prevalent among preterm newborns (86.9%) than term ones (59.5%) as shown from the cross tabulation (Table 5). Regarding newborns with perinatal asphyxia, it was found that all the perinatally asphyxiated newborns were hypothermic which may be due to the fact that these newborns can’t generate heat as they are in scarce of oxygen (hypoxia) [Fig. 3]. From the scatter plot of their exact body temperature, it can also be easily understood that nearly all the perinatally asphyxiated newborns were moderately hypothermic with a median body temperature of 34.1 °C and IQR of 2.35 °C (Table 6).

**Table 5:** Cross tabulation of gestational age by thermal status among newborns that were delivered at public health institutions of Harar city, Eastern Ethiopia, 2018

		Thermal status		Total
		Hypothermic	Non hypothermic	
Gestational age	Preterm	86	13	99
	Term	181	123	304
Total		267	136	403

**Table 6:** Median and IQR of the exact body temperature for perinatally asphyxiated newborns that were delivered at public health institutions of Harar city, Eastern Ethiopia, 2018

N	Valid	25
	Missing	0
Median		34.10
Percentiles	25	32.85
	50	34.10
	75	35.20

* IQR = 35.20 °C-32.85 °C = 2.35 °C

### Predictors of neonatal hypothermia

Newborns who weren’t made in skin to skin contact with their mothers after delivery were 2.9 times more likely to be hypothermic as compared to those who had skin to skin contact (AOR = 2.87,95% CI: 1.48, 5.57). Newborns who didn’t wear cap were 2 times more likely to be hypothermic when compared to those who were dressed with cap (AOR = 2.10, 95% CI: 1.17, 3.76). Newborns who weren’t warmly transported from one unit (delivery) to the other (postnatal unit or NICU) were 3.2 times more likely to be hypothermic when compared to those warmly transported ones (AOR = 3.18, 95% CI: 1.84, 5.48). Those newborns who were born to mothers having obstetric complication were 2.4 times more likely to be hypothermic as compared to those born to mothers without any obstetric complication (AOR = 2.42, 95% CI: 1.28, 4.57). Preterm newborns were 3.4 times more likely to be hypothermic as compared to term ones (AOR = 3.37, 95% CI: 1.53, 7.44). Moreover, newborns with health problems were about 4 times more likely to be hypothermic in relative to those who didn’t have any problem (AOR = 4.24, 95% CI: 1.92, 9.34) (Table 7).

**Table 7:** Predictors of neonatal hypothermia among newborns that were born at public health institutions of Harar city, Eastern Ethiopia, 2018

Variable(n = 403)	Hypothermic (267)	Non hypothermic (136)	COR (95%CI)	AOR (95%CI)
	F (%)	F (%)		
Skin to skin contact				
Yes	31 (30.4%)	71 (69.6%)	1.0	1.0
No	236 (78.4%)	65 (21.6%)	8.32 (5.03, 13.76)	2.87 (1.48, 5.57) ***
Wearing cap				
Yes	112 (51.9%)	104 (48.1%)	1.0	1.0
No	155 (82.9%)	32 (17.1%)	4.50 (2.83, 7.16)	2.10 (1.17, 3.76)*
Proper wrapping				
Yes	99 (50.5%)	97 (49.5%)	1.0	1.0
No	168 (81.2%)	39 (18.8%)	4.22 (2.70, 6.60)	1.48 (0.83, 2.64)
Early breast feeding				
Yes	179 (62.4%)	108 (37.6%)	1.0	1.0
No	88 (75.9%)	28 (24.1%)	1.90 (1.16, 3.09)	1.63 (0.88, 2.99)
Warm transportation				
Yes	69 (45.4%)	83 (54.6%)	1.0	1.0
No	198 (78.9%)	53 (21.1%)	4.49 (2.89, 6.98)	3.18 (1.84, 5.48) ***
Obstetric complication				
Yes	100 (84%)	19 (16%)	3.69 (2.14, 6.36)	2.42 (1.28, 4.57) **
No	167 (58.8%)	117 (41.2%)	1.0	1.0
Birth weight (Kg)				
< 2.5(LBW)	69 (85.2%)	12 (14.8%)	3.60 (1.88, 6.92)	1.20 (0.51, 2.82)
≥ 2.5 (NBW)	198 (61.5%)	124 (38.5%)	1.0	1.0
Gestational age (weeks)				
< 37(pre-term)	86 (86.9%)	13 (13.1%)	4.50 (2.40, 8.41)	3.37 (1.53, 7.44) ***
≥ 37(Term)	181 (59.5%)	123 (40.5%)	1.0	1.0
Neonatal health problem				
Yes	94 (90.4%)	10 (9.6%)	6.85 (3.43, 13.67)	4.24 (1.92, 9.34) ***
No	173 (57.9%)	126 (42.1%)	1.0	1.0

*Significant at 0.013, **significant at 0.007 and *** significant at < 0.004

## Discussion

The prevalence of neonatal hypothermia among newborns in the study area was 66.3% which was almost similar with studies conducted in Nigeria (62%), Addis Ababa (64%), Bahir Dar, Northwest Ethiopia (67%) and Gondar, Northwest Ethiopia (69.8%). This prevalence was lower than studies conducted at Babol, Iran (84.5%), Zimbabwe (85%), and Uganda (83%), but higher than studies conducted in Tehran, Islamic Republic of Iran (53.3%), South Africa (21%), Guinea Bissau (8.1%) and Tanzania (22%) [10, 16, 22, 24–26]. This variation might be due to differences in the temperature measuring instrument, study design, temperature measurement site, sociocultural and ecological factors between the study areas.

This study revealed that newborns with health problems were about 4 times more likely to be hypothermic in relative to those who didn’t have. This might be due to the fact that newborns with problems are less likely to breastfeed effectively which makes them to be hypoglycemic that in turn results in hypothermia. It may also be due to another fact that newborns with problems can’t take up and utilize oxygen effectively for heat energy production. This finding is almost consistent with the study held at Gondar University Hospital, Northern Ethiopia [16].

Moreover, the study revealed that preterm newborns were 3.4 times more likely to be hypothermic as compared to term ones. The possible reason may be due to the fact that preterm newborns have thinner and more immature skin that increases heat loss through radiation, poor hypothalamic control of their body temperature, lack of efficient neural mechanisms for temperature control by shivering, decreased glycogen stores, decreased subcutaneous fat for thermal insulation, less brown fat tissue, decreased ability to breastfeed effectively and decreased ability to regulate their body temperature through non-shivering thermogenesis [1, 8, 11, 23]. This finding was congruent with a study done at Addis Ababa, Ethiopia and Southern Nepal [21, 24].

Those newborns that were born to mothers with obstetric complication were 2.4 times more likely to be hypothermic as compared to those born to mothers without any obstetric complication. This could be due to newborns that were born to mothers with obstetric complication usually have health problems (respiratory distress, perinatal asphyxia, hypoglycemia, etc.). Moreover, newborns of mothers with obstetric complication are often born preterm and/ low birth weight [8, 27]. This finding is consistent with a Californian cross-sectional data analysis [20].

Newborns who didn’t wear cap were 2 times more likely to be hypothermic when compared to those who were dressed with cap and it was comparable with the study held at southern Nepal [21]. This could be due to their large head with open fontanels and sutures which contribute to almost 25% of neonatal heat loss unless dressed with cap [1].

Newborns that weren’t made in skin to skin contact with their mothers after delivery were almost 3 times more likely to be hypothermic as compared to those who had skin to skin contact. This may be due to the warm chain principle that there is no transfer of heat from mother to the newborn unless the newborn is in direct contact with its mother’s skin. Skin to skin contact is more effective than incubator care for re-warming the newborn. It may also be due to the maternal chest and abdominal movement that stimulates the newborn for enhanced breathing which improves heat generation through oxidative phosphorylation [1, 8]. This finding is almost consistent with Ethiopian studies conducted at Addis Ababa (AOR = 4.39, 95% CI: 2.38, 8.11) and Gondar, North west Ethiopia (AOR = 2.81, 95%CI: 1.40, 5.66) [16, 24]. On the other hand, the prevalence of hypothermia among newborns of the study area that weren’t made in skin to skin contact (78.4%) was lower than a Ugandan study that showed the prevalence of hypothermia being 87% among newborns without skin to skin contact [22]. This discrepancy might be due to differences in the thermometric site, type of thermometer, maternal awareness of the advantages of skin to skin contact and study period (seasonal variations).

Newborns that weren’t warmly transported from delivery unit to postnatal or neonatal intensive care unit were 3.2 times more likely to be hypothermic as compared to those warmly transported ones. This may be due to the convective and radiant heat loss when newborns aren’t kept in contact with their mothers’ skin during transportation [1, 8].

### Limitation of the study

The study lacks support of qualitative data. As this study was conducted only in the summer season, it couldn’t show the significance of seasonal variation for neonatal hypothermia. Moreover, the results may not be representative of the entire newborns in Ethiopia due to a small sample size consideration in this study. Besides, the impact of air temperature of the delivery and postnatal units wasn’t considered due to absence of inbuilt thermostat and humidity control in the studied public health institutions. The study also shares drawbacks of a cross-sectional design.

## Conclusion

The prevalence of neonatal hypothermia in the study area was relatively high. Furthermore, it was found that wearing cap; having skin to skin contact and warm intra-facility newborn transportation were significantly associated with decreased odds of hypothermia whereas neonatal health problems, prematurity and born to mothers with obstetric complication were associated with increased odds of hypothermia.

### Based on the study finding, the following public health measures were recommended

#### For maternity and neonatal health care providers

Neonatal care providers should adhere to the routine practice of warm chain by giving most prioritized attention to newborns with health problems, preterm newborns and those born to mothers with obstetric complication. Mothers should also be oriented of thermal care during their antenatal care, while they are at labor, delivery and postnatal unit.

#### For the public health institutions

Every delivery room should have its own thermostat and humidity control so that labor and delivery personnel can adjust the thermostat as needed for any delivery. Delivery room temperature and humidity should be documented at the time of each delivery. Each newborn’s temperature should also be measured and recorded as soon as possible after birth and then every 10–15 min. The postnatal and neonatal intensive care units should be suitably arranged to the delivery unit so that mothers can’t be in difficulty of skin to skin contact during intra-facility transportation. The practice of warm chain should also be supervised on a regular basis.

#### For researchers

The authors recommend a follow up study supplemented with qualitative data mainly to identify the significance of some factors like seasonal variation.

## Data Availability

Data will be available upon request from the corresponding author.

## Abbreviations

AOR: Adjusted Odds Ratio
APGAR: Appearance, Pulse, Gremace, Activity, Respiration
C/S: Caesarean Section
CI: Confidence Interval
COR: Crude Odds Ratio
CPR: Cardio Pulmonary Resuscitation
GA: Gestational Age
LBW: Low Birth Weight
NBW: Normal Birth Weight
NICU: Neonatal Intensive Care Unit
SVD: Spontaneous Vaginal Delivery
WHO: World Health Organization
